# Cognitive, relational and task crafting: Spanish adaptation and analysis of psychometric properties of the Job Crafting Questionnaire

**DOI:** 10.1371/journal.pone.0223539

**Published:** 2019-10-07

**Authors:** Onintze Letona-Ibañez, Maria Carrasco, Silvia Martinez-Rodriguez, Alejandro Amillano, Nuria Ortiz-Marques

**Affiliations:** 1 Department of Social Psychology and Development, Faculty of Psychology and Education, University of Deusto, Bilbao, Spain; 2 Department of Personality, Assessment and Psychological Treatment, Faculty of Psychology and Education, University of Deusto, Bilbao, Spain; 3 Department of Social Pedagogy and Diversity, Faculty of Psychology and Education, University of Deusto, Bilbao, Spain; University of Lleida, SPAIN

## Abstract

Even though classic job design theories have evolved over the years and become more focused on employees’ ability to autonomously change their job characteristics, tools to assess job crafting are still limited. The purpose of this study was to analyze the psychometric properties of the Spanish version of the Job Crafting Questionnaire (JCQ), taking into account the valuable contribution made by Wrzesniewski and Dutton’s model to the understanding of the job crafting concept. The total sample consisted of 768 employees (participants’ mean age was 41.63 and 49.7% of them were women). The sample was randomly divided into two halves in order to conduct two factor analyses (Exploratory Factor Analysis and Confirmatory Factor Analysis). Concurrent and convergent validity was assessed by computing correlations with validated questionnaires for measuring job crafting (Job Crafting Scale, JCS), engagement (Utrecht Work Engagement Scale, UWES-9) and job burnout (Maslach Burnout Inventory-General Survey, MBI-GS). The results indicated a high level of internal consistency (Cronbach’s alpha = .880) which was similar to the original scale, and provided a good fit to the three-dimensional model tested. Appropriate evidence of construct validity was also shown (r = .45 with total JCS; r = .52 with total UWES-9 and r-values between -.33 and .45 with MBI dimensions). The results confirmed that the Spanish translation of the JCQ is a suitable tool for measuring job crafting and enabling practitioners and researchers to further expand the existing knowledge of this concept.

## Introduction

As the wellbeing of the individual is becoming increasingly important to organizations, the classic job design theories are evolving to strengthen their focus on employees’ ability to autonomously change their job characteristics. This is known as job crafting, a concept initially proposed by Wrzesniewski and Dutton [1]. They sought to move away from how work contexts had been conceptualized, and proposed that employees should be able to change some aspects of their work, even in the most routine and restricted jobs. Their definition of job crafting was active behavior whereby employees attempt to make physical and/or cognitive changes to the tasks or personal relationships in their work.

Wrzesniewski and Dutton [1] identified three forms of job crafting: task crafting (which relates to the tasks or work activities that an individual could change in the number, scope or type of job tasks performed); relational crafting (which refers to the changes that an employee can make to the quantity and/or quality of interactions with others on the job); and cognitive crafting (which involves cognitively altering how one sees one’s job either as a set of discrete tasks or as an integrated whole with a meaning of its own). In a later qualitative study, Berg, Wrzesniewski and Dutton [2] were able to further describe what workers understood by each of these dimensions of job crafting.

Some years later, Tims and Bakker [3] resumed the task of defining job crafting and attempted to complement the model presented by their predecessors, which they deemed to be too general in its approach. They incorporated the concept of job crafting to the Job Demands-Resources (JD-R) model [4, 5], which provides a reference framework to understand an individual’s work environment by categorizing its components as demands (e.g. time pressure, high workload, shift work) and resources (e.g. feedback, job control, supervisor support).

Tims and Bakker [3] initially posited three categories or forms of job crafting to specify the aspects that individuals try to change in their job. First, “increasing job resources”, which refers to those resources that can be mobilized by employees to meet the demands of their work. Some examples include having greater autonomy and high levels of social support. Second, “increasing challenging job demands”, relates to the changes that employees can make if they feel that their work does not provide them with sufficient opportunities to use all their skills and expertise. These types of demands are related to positive work outcomes if employees feel that they have sufficient resources to meet their job demands. Third, “decreasing hindering job demands”, has to do with the individual’s attempt to reduce those tasks deemed to be demanding or to hamper positive outcomes. Subsequently the same authors subdivided “increasing job resources” into two categories and argued there are two specific types of job resources: structural job resources and social job resources [6], thereby building a four-dimensional model.

While different conceptualizations or proposals can be found that seek to complement the models described above [7–9], those by Wrzesniewski and Dutton [1] and Tims and Bakker [3] are the main theoretical approaches to the concept of job crafting. In both approaches job crafting involves engaging in a set of activities mainly intended to obtain individual benefits, although they differ in the specific objectives sought by employees when they make changes to their job.

Wrzesniewski and Dutton [1] proposed that the ultimate objective of job crafting is to create alterations in the meaning of one’s work and personal identity. Making changes to their job enables employees to experience their work differently, and therefore, to reframe the meaning they attribute to it. They may also modify their work interactions and how they contribute to their objective of reinforcing or sustaining their identity.

The conceptualization developed by Tims and Bakker [3], in contrast, did not consider the potential cognitive changes that employees can make in order to assign a different, more positive meaning to their job. They proposed that job crafting activities are aimed at adjusting the individual’s perceived work demands and resources (person-job fit).

Three studies recently discussed these conceptual differences and highlighted the need to propose new ways to integrate these two approaches. Bruning and Campion [7] suggested a new taxonomic model called the “role-resource approach-avoidance model of job crafting”, in which they linked the role-based perspective to Wrzesniewski and Dutton´s [1] approach and described job crafting as an individual’s attempt to make their job role responsive to their personal needs. They also related the resource-based perspective to Tims and Bakker model [3], and regarded job crafting as a strategy through which the individual finds resources with which to respond to their job demands. The review by Zhang and Parker [8] proposed the existence of a three-level conceptualization which, on the one hand, reinforced the idea that the two main models of job crafting indicate certain differences in approach, and on the other hand, brought a separate dimension to cognitive crafting. Taking into account the previous studies’ contributions, Lichtenthaler and Fischbach [9] proposed a new approach that also aimed to unite the two main perspectives of job crafting. They introduced the notions of promotion-focused job crafting (referred to the dimensions of an individual’s learning and development objectives as challenging job demands increase, or cognitive crafting), and prevention-focused job crafting (referred to the dimensions that seek to reduce work or relational demands).

The conceptualization of job crafting is therefore a relatively new notion within organizational psychology, and has been analyzed in numerous studies from differing perspectives. In general terms, however, research on job crafting has been related to important variables linked to the organizational context and the wellbeing of employees, including engagement, job satisfaction, job performance and low burnout levels, among others.

A longitudinal study by Tims, Bakker and Derks [10] found that the increase in work and social resources and the search for challenging job demands predicted the increase in work engagement and job satisfaction, while also reducing burnout levels. Along the same lines, Vogt, Hakanen, Brauchli, Jenny and Bauer [11] showed that an increase in job crafting resulted in an increase in long-term work engagement levels, as well as helping boost personal resources such as employees’ Positive Psychological Capital. A relationship has also been shown between job crafting and work engagement in teams [12]. A study recently conducted on a sample of Finnish dentists indicated that job crafting moderated the effect that some of the most frequent work demands among this population had on burnout, as stress levels were reduced [13]. These results have also been shown to be consistent in different cultural settings. For example, the study by Sakuraya et al. [14] found that job crafting was related to greater levels of engagement among a sample of Japanese employees.

The relationship between job crafting and job performance has also been analyzed in several studies. Lichtenthaler and Fischbach [15] recently found that there is a strong relationship between some of the dimensions of job crafting and the performance of workers as assessed by their superiors. Other proactive characteristics, such as intrinsic motivation related to work and organizational citizenship behavior (OCB), have also been related to job crafting [16].

Rudolph, Katz, Lavigne and Zacher [17] conducted a meta-analytical study that reviewed a total of 122 independent samples, and concluded that job crafting was positively related to variables that included job satisfaction, work engagement and work performance, and negatively related to job strain.

### Measuring Job Crafting

Some of the first tools to evaluate job crafting were proposed by Ghitulescu [18] and by Leana, Appelbaum and Shevchuk [19], based on the theoretical framework developed by Wrzesniewski and Dutton [1]. These proposals were highly specific to their populations of interest (manufacturers and teachers, respectively) and subsequently some authors have expressed their difficulty in expanding these instruments to more general contexts [18].

The model developed by Tims and Bakker [3] has also been used as a framework to build some of the tools to measure job crafting that can be found in the literature, including the questionnaire adapted by Petrou, Demerouti, Peeters, Schaufeli and Hetland [20], the Job Crafting Questionnaire (JCRQ) proposed by Nielsen and Abildgaard [21] and the Job Crafting Scale [6]. The first two questionnaires mentioned above were adapted from the Job Crafting Scale [6].

Based on its underlying theoretical framework, the Job Crafting Scale (JCS) [6] initially relied on three factors (the same as in the model proposed by Tims and Bakker) [3]. They were measured by using 42 items and this structure was first tested on a sample of 375 people. After conducting a confirmatory analysis on two samples different from the initial one (415 and 201 participants, respectively), the scale was built on 4 dimensions: increasing structural job resources, decreasing hindering job demands, increasing social job resources, and increasing challenging job demands. To measure these dimensions, 21 items were proposed, using a 5-point Likert scale (from 1 = never to 5 = often). The results from Cronbach’s alpha for each of the dimensions (.82, .79, .77 and .75, respectively) indicated appropriate levels of reliability.

Another scale used to measure this concept is the Job Crafting Questionnaire (JCQ), developed by Slemp and Vella-Brodick [16]. Unlike the instruments described above, it is based on the initial theoretical model proposed by Wrzesniewski and Dutton [1]. The process to create the JCQ was completed by using a sample of 334 employees. Initially 27 items were proposed in order to measure the three dimensions of job crafting: task crafting, cognitive crafting and relational crafting. Four of these items were adapted from the scale by Leana et al. [19]. After a pilot study, the scale was reduced to 21 items (7 for each dimension). Participants were asked to respond using a 6-point Likert scale, from 1 (hardly ever) to 6 (very often). Following exploratory and confirmatory analyses on two independent samples, a three-dimension structure with 15 items (5 items for each of the dimensions) was confirmed. Cronbach’s alpha coefficients were calculated for each of the three dimensions, and were all above .70 (.87, .89 and .83, respectively), indicating good internal consistency. This instrument has recently been validated in German, maintaining the original factorial structure and showing a good psychometric adjustment [22].

As far as we know, only two of the instruments mentioned here have been validated on the Spanish population (both of which were recently published). This means that there is increasing interest in this construct within positive organizational psychology, including in Spain. The Job Crafting Questionnaire (JCRQ) developed by Nielsen and Abildgaard [21], an adaptation of the JCS specifically focused on blue-collar workers, consists of 5 dimensions (increasing challenging job demands, decreasing social job demands, increasing social job resources, increasing quantitative job demands and decreasing hindering job demands) and was validated by Nielsen, Antino, Sanz-Vergel and Rodríguez-Muñoz [23] in a study conducted on 164 employees from different work sectors. In addition, a Spanish version of the JCS [6] was recently published by Bakker, Ficapal-Cusí, Torrent-Sellens, Boada-Grau and Hontangas-Beltrán [24]. This scale was validated on a sample of 896 employees from the industry and services sectors. It confirmed the 4-factor structure proposed by the original authors, and obtained Cronbach alpha coefficients that ranged from .70 to .79.

The job crafting variable has been largely measured within the Job Demands and Resources theory, and has been evaluated by using the questionnaire developed by Tims, Bakker and Derks [6]. However, as several authors have noted, including Bruning and Campion [7] and Rudolph et al., [17], this model excludes the cognitive dimension of job crafting contained in the model by Wrzesniewski and Dutton [1]. Moreover, there are only a few instruments in Spanish based on the initial model. Therefore, there is a need to have an instrument in Spanish that measures the original concept of job crafting and takes into account the cognitive dimension of this variable. This will help to further the knowledge currently available about this construct, as some authors have raised concerns about the potential conceptual overlap between the existing proposals. Also, the relationship between the dimension related to cognitive job crafting advocated by Wrzesniewski and Dutton [1] and other positive variables is even more unclear and still largely unknown [17]. Considering that organizations, the frame of reference for this study, are contexts directly influenced by cultural characteristics, it is important for them to have access to instruments that have been adapted and take into consideration, not only the adjustment of the psychometric properties, but also the cultural appropriateness of the tool. This perspective enhances the standards of the adaptation process and of cross-cultural research [25].

This study is focused on the Spanish translation of the Job Crafting Questionnaire developed by Slemp and Vella-Brodick [16] and on testing its validity. Taking into account the contribution made by the model created by Wrzesniewski and Dutton to the understanding of the job crafting concept, it seems essential to provide a tool that can give further insight into job crafting than that drawn from the tools currently available in Spanish.

## Materials and methods

### Participants

The sample consisted of 768 employees resident in Spain; 50.1% were men and 49.7% were women, with a mean age of 41.63 (SD = 9.753; age range: 23–71). Some 86.3% of the participants held a (graduate or postgraduate) degree. A large percentage of them were in managerial positions (30.9%) and 34.1% worked as scholars, scientific experts, or professionals (in professions related to health and education). Of these, 71.9% had a permanent contract, 87.1% worked full-time, and 71.9% reported that they had a mid-to-high salary. Some 37.5% stated that they had been employed for over 20 years, 35.8% that they had been employed between 10 and 20 years and 24.5% that they had been in their current job for more than 10 years.

### Materials

Two different instruments (based on different theoretical models) were used in this study to evaluate job crafting. The Job Crafting Scale provided data with which to check the concurrent validity of the Job Crafting Questionnaire. Engagement and burnout variables were also used in order to examine the convergent validity of the Job Crafting Questionnaire.

Job Crafting (as based on the theoretical background by Wrzesniewski and Dutton [1]) was measured using the version of the Job Crafting Questionnaire adapted to Spanish [16], which consists of 15 items that measure three dimensions: task crafting (e.g., “Change the scope or types of tasks that you complete at work”), relational crafting (e.g., “Make an effort to get to know people well at work”) and cognitive crafting (e.g., “Remind yourself of the importance of your work for the broader community”) on a 6-point Likert-type scale (1 = hardly ever, 6 = very often) (see Appendix 1).

Job Crafting (as based on the theoretical background by Tims and Bakker [3]) was assessed by using the version of the Job Crafting Scale [24] adapted to Spanish, which consists of 4 dimensions (increasing structural job resources, decreasing hindering job demands, increasing social job resources and increasing challenging job demands) measured by 21 items on a 5-point Likert-type scale (1 = never, 5 = often) (e.g., “I make sure that I use my capacities to the fullest”).

Work Engagement was assessed by using the Spanish version of the Utrecht Work Engagement Scale-9 (UWES-9 [26]), translated by the same authors. It measures work engagement along three dimensions: vigor, dedication, and absorption, each containing three items, which were scored on a 7-point Likert-type scale (1 = never, 7 = always) (e.g., “At my work, I feel bursting with energy”).

Job Burnout was measured using the Spanish translation of the Maslach Burnout Inventory-General Survey (MBI-GS [27]) carried out by Moreno-Jiménez, Rodríguez-Carvajal and Escobar [28], which contains 16 items by which the three dimensions of this concept (emotional exhaustion, cynicism and personal efficacy) are measured on a 7-point Likert-type scale (ranging from 0 = never to 6 = every day) (e.g., “I feel emotionally drained from my work”). High scores in exhaustion and cynicism and low scores in personal efficacy correspond to high degrees of burnout.

### Procedure

Participants were contacted through acquaintances of the researchers and a jobseeking-related social network, in order to make random contact with working people. In both cases, people were first invited to take part in the survey, the objective of which was briefly explained to them. If they accepted this invitation, they were sent a link to the online questionnaire, where they found an informed consent form to participate in the survey, and a more detailed explanation about its objectives and what it involved. This sampling procedure and the entire procedure used in the survey were approved by the Ethics Committee of the University of Deusto (Ref. ETK-23/17-18).

#### Translation process

The following translation and back-translation method was used to translate the JCQ [29] into Spanish, following the directions given by Brislin [30]. Two bilingual lecturers with vast experience in translating texts from the field of psychology assisted in the process, and the final version of the translated questionnaire was revised by two specialist researchers. The aim of the experts was to preserve the conceptual content of the items, while ensuring that the language was culturally suitable for Spanish speakers. Once the translation had been verified, the questionnaire was sent to the participants using an online survey in which sociodemographic data was also requested.

### Data analysis

The response rates, mean, standard deviation, asymmetry and kurtosis were calculated for each of the items in order to analyze the descriptive data of the sample regarding the JCQ. Internal consistency was assessed by calculating the correlation between each item and the scale total, the total Cronbach’s alpha if an item was deleted from the questionnaire, and for the full questionnaire. A descriptive analysis of the sample was also carried out, analyzing differences related to gender, age, level of education and years of working life.

The total sample was randomly divided into two halves in order to conduct two factor analyses. An Exploratory Factor Analysis (EFA) was carried out on the first sample (n = 386). The suitability of the correlation matrix was verified to ensure that it was factorized on the basis of the Kaiser-Meyer-Olkin test and the Bartlett sphericity test. Parallel Analysis (PA) [31] and Minimum Average Partial Method (MAP) [32] tests were carried out as extraction criteria for the advisable number of factors according to the configuration of the correlation matrix. The multivariate normality was also analyzed with the Mardia test [33]. These tests were conducted using FACTOR software [34].

In order to test the validity of the instrument on the basis of the theoretical model proposed, a Confirmatory Factor Analysis (CFA) was carried out on the second sample (n = 382) using EQS [35]. A non-weighted least squares approach and robust methods were used, taking into account the measuring scale and the fact that the data did not follow multivariate normal distribution [36, 37]. Because of the sensitivity of the chi-square test to sample size [38], other goodness-of-fit indexes were taken into account [39], including Root Mean Square Error of Approximation (RMSEA), which has cutoff values for a good fit <0.05; and an adequate fit <0.08. The Goodness-of-Fit Index (GFI) and the Comparative Fit Index (CFI), with values of >0.90, Chi-square/degrees of freedom ratio (χ2/df) with acceptable values below 3.0, and the Akaike Information Criteria (AIC) were eligible to compare the models with different estimated parameters for which lower values would indicate higher parsimony.

**Fig 1:**
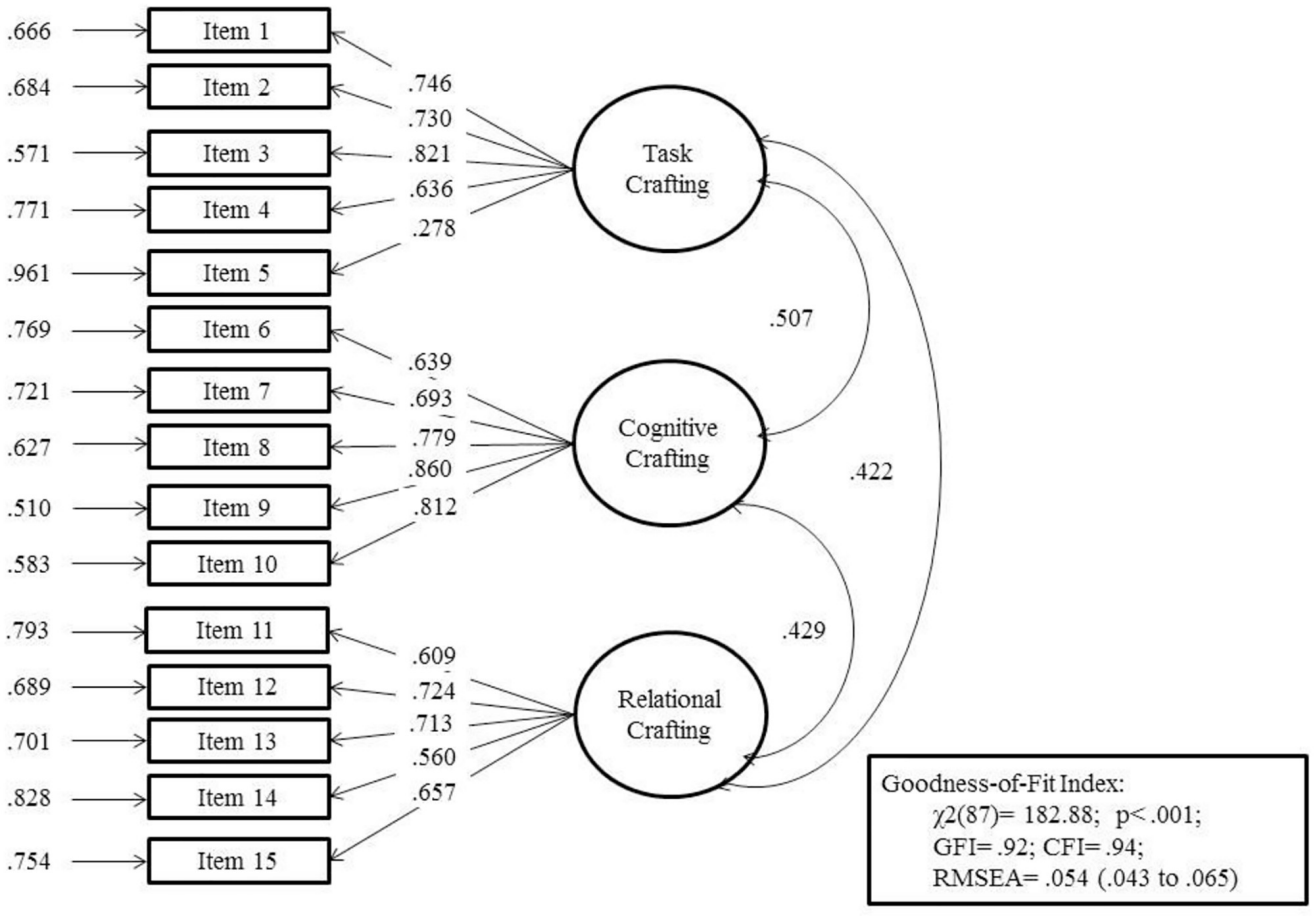
3-factor structural model with 15 items (n = 382). Correlations between factors are statistically significant (p<.05).

The final structural model has been represented (Fig 1) by indicating factor weights, correlation between factors and fitness indexes. The composite reliability and the average variance extracted were also calculated for each factor.

Concurrent and convergent validity was examined using Spearman’s rank correlation in SPSS (Statistical Package for Social Sciences) for Windows, version 23.0.

All statistical tests were significant at p<.05 (α = 5%).

## Results

### Descriptive statistics

Table 1 shows the main descriptive and internal consistency data related to the 15 items in the Job Crafting Questionnaire on the total sample of this survey (N = 768). The mean score for the questionnaire was 4.57 (SD = 0.73) on a scale from 1 to 6, which means that the employees who participated in the study perceived medium-to-high levels of job crafting.

**Table 1:** Descriptive statistics and internal consistency of the Job Crafting Questionnaire.

	Response distribution percentage (n = 768)						Descriptive statistics				Internal consistency	
No.	1	2	3	4	5	6	M	SD	Sk	Kur	r	Alpha
1	0.8	1.7	3.4	19.3	40.1	34.8	5.01	0.97	-1.17	1.94	.595	.873
2	1.2	6.0	7.7	27.9	39.6	17.7	4.52	1.12	-0.82	0.49	.521	.876
3	0.9	3.0	6.4	22.4	43.2	24.1	4.76	1.04	-0.98	1.16	.571	.874
4	1.8	3.1	8.1	16.9	42.2	27.9	4.78	1.13	-1.14	1.23	.526	.875
5	1.2	2.2	8.1	23.6	39.5	25.5	4.74	1.06	-0.90	0.90	.365	.881
6	3.8	7.0	9.1	17.6	30.2	32.3	4.60	1.39	-0.93	0.04	.600	.873
7	2.7	7.2	12.0	20.8	31.0	26.3	4.49	1.33	-0.74	-0.19	.685	.868
8	5.3	9.5	12.2	22.8	27.2	22.9	4.26	1.45	-0.61	-0.51	.709	.867
9	1.4	5.6	11.6	20.6	33.3	27.5	4.61	1.23	-0.77	-0.03	.701	.868
10	1.7	7.0	10.2	16.7	36.2	28.3	4.63	1.27	-0.89	0.06	.658	.870
11	0.3	2.5	7.2	18.9	34.5	36.7	4.95	1.05	-0.92	0.41	.538	.875
12	9.0	13.7	14.6	21.4	24.9	16.5	3.89	1.55	-0.35	-0.95	.590	.875
13	6.9	12.0	13.0	18.0	29.0	21.1	4.14	1.53	-0.53	-0.80	.593	.875
14	4.2	7.8	10.8	24.6	29.6	23.0	4.37	1.37	-0.71	-0.19	.584	.874
15	0.8	4.7	6.4	18.5	37.6	32.0	4.84	1.13	-1.04	0.75	.538	.875
*Total*							4.57	0.73	-0.59	0.25		.880

M = mean; SD = standard deviation; Sk = skewness; Kur = kurtosis; r = correlation between item score and total scale score; Alpha = coefficient if an item is removed

Items 1 and 4 yielded slightly negative asymmetry scores (-1.17, -1.14) and kurtosis (1.94 and 1.23). Item 3 had slight kurtosis (1.16), and item 15 yielded a very slight negative asymmetry score (-1.04). Asymmetry and kurtosis values were moderate for the remaining items in the questionnaire (between -1 and +1). Item-total correlation values were greater than .40, except for item 5 (r = .36). If any of the items were removed, it would not enhance the scale reliability (α = .880).

The levels obtained in the different variables were analyzed based on the basic socio-demographic characteristics of the sample: age, gender and years of working life.

Significant differences related to gender were found in the dimensions of cognitive crafting (t(766) = -2.987 p<.005, d = 0.215) and relational crafting (t(766) = -2.299 p<.05, d = 0.022), as well as for the total job crafting score evaluated with the JCQ (t(766) = -2.336 p<.05, d = 0.016). In all cases the scores were higher for women. Age-related differences were also found for cognitive (F(2, 765) = 4.728, p<.01, η^2^ = .012) and relational crafting F(2, 765) = 3.074, p<.05, η^2^ = .008). For cognitive crafting, people over 55 showed the highest scores compared with younger participants. But as far as relational crafting was concerned, the youngest people (<34 years) obtained the highest scores compared to workers between 35 and 54 years of age. Age-related differences were also found in two of the burnout dimensions: cynicism (F(2, 751) = 4.168, p<.05, η^2^ = .011) and professional efficacy (F(2, 744) = 4.816, p<.01, η^2^ = .013). Workers under the age of 34 showed higher levels of cynicism than people over the age of 55. Similarly, differences were found in engagement levels (F(2, 754) = 6.448, p<.01, η^2^ = .017). It is interesting to note that people over the age of 55 showed the highest levels of professional effectiveness, engagement and cognitive crafting.

When taking into account the total years of working life, significant differences were found in the levels of cynicism (F(3, 750) = 3.571, p<.05, η^2^ = .014), professional efficacy (F(3,743) = 6.456, p<.001, η^2^ = .025), engagement (F(3, 753) = 4.258, p<.01, η^2^ = .017) and the task crafting dimension of the JCQ (F(3, 764) = 5.322, p<.01, η^2^ = .020). For professional efficiency, engagement and task crafting, those who had been working for more than 20 years obtained significantly higher scores. However, those who had been working for less than 5 years showed higher levels of cynicism than those who had been working for more than 20 years.

### Factor analysis

An EFA was carried out on the first sample (n = 386). Mardia’s coefficient was high (33.97), indicating multivariate non‐normality, and therefore factor analyses were performed based on a polychoric correlation matrix. The determinant value of the matrix (0.001), the Kaiser-Meyer-Olkin test (KMO = .86) and Barlett sphericity test (χ2(105) = 2543.2; p< .001) meant that the matrix could be factored. Table 2 shows the results of the EFA for a three-factor model, as proposed by the authors of the questionnaire [17]. The items had a similar distribution to that in the original questionnaire (F1: items 1 to 4; F2: items 6 to 10; F3: items 11 to 15) and exhibited factor loadings greater than .40 (ranging from .40 to .93), except for item 5. F1 showed a high eigenvalue compared with the eigenvalues of the other two factors (6.19, 1.68 and 1.49, respectively) and jointly accounted for 62% of the variance. Results of the PA suggested that one factor should be retained with factor loadings greater than .40 (ranging from .46 to .72) and the MAP test indicated that two factors should be retained (F1: items 1 to 10; F2: items 11 to 15) with factor loadings greater than .40 (ranging from .40 to .87).

**Table 2:** Exploratory factor analysis (n = 386).

	FL			
	F1	F2	F3	h^2^
Eigenvalue	6.19	1.68	1.49	
Explained variance	0.41	0.11	0.09	
Alpha	.758	.868	.790	
No.				
1	.66			.551
2	.83			.607
3	.78			.599
4	.64			.453
5	.28			.231
6		.69		.492
7		.67		.572
8		.63		.558
9		.93		.768
10		.85		.672
11			.58	.447
12			.84	.619
13			.78	.596
14			.40	.422
15			.67	.499

FL = factor loading; h2 = communality

A Confirmatory Factor Analysis (CFA) was carried out on the second sample (n = 382). First, the three-factor model (M1) proposed by the authors of the scale was tested including the 15 items contained in the JCQ. As shown in Table 3, this model shows good fit indexes, despite the fact that Satorra-Bentler Scaled Chi-square was statistically significant. The Wald Test did not suggest that any parameters be removed, although item 5 had a lower factorial load than the rest of items, as it approached .30. In addition, the scale reliability did not improve after its elimination, so it was decided to maintain the item consistent with the model presented by the authors of the scale. According to the PA results, which suggested using a single factor, and in order to analyze the an overall job crafting index, three other models were tested: a single factor model (M2), a three first-order factors and one second-order factor model (M3), and a bifactorial model (M4) in which each of the items simultaneously loaded on a single general factor and three first-order factors, which refer to all three dimensions. Results showed a worse fit than the first model tested. Finally, consistent with the MAP results, a two factors model was tested (M5) in which, unlike the bifactorial model, the items loaded onto two first-order factors. But the latter also showed a lack of fit.

**Table 3:** Goodness-of-fit indexes of the models tested.

	χ^2^_SB_	df	*p*	χ^2^/df	GFI	CFI	RMSEA	95% Confidence Interval	AIC
M1	182.88	87	<.001	2.10	.92	.94	.054	(.043 to .065)	8.88
M2	674.98	90	<.001	7.49	.70	.63	.131	(.121 to .140)	494.98
M3	249.24	32	<.001	7.78	.80	.80	.133	(.118 to .149)	185.24
M4	305.25	90	<.001	3.39	.86	.86	.079	(.069 to .089)	125.25
M5	448.14	89	<.001	5.03	.78	.77	.103	(.093 to .112)	270.14

M1: three factor model; M2: single factor model; M3: three first-order factors and one second-order factor model; M4: bifactorial model; M5: two factors model; χ^2^_SB_: Satorra-Bentler Scaled Chi-square; df: degree of freedom; *p*: probability; χ^2^/df: Chi-square/degree of freedom ratio; GFI: The Goodness-of-Fit Index; CFI: the Comparative Fit Index; RMSEA: Root Mean Squared Error of Approximation; AIC: Akaike Information Criterion

Fig 1 shows the structural three-factor model with 15 items. The three factors demonstrated adequate composite reliability (.73, .81 and .73, respectively) and average variance extracted scores ranging from 0.42 to 0.57 (0.44, 0.57 and 0.42, respectively).

### Construct validity: Concurrent and convergent validity

Evidence of construct validity for the Spanish version of JCQ was supported by the results from the aforementioned confirmatory and exploratory factor analyses, as well as by the correlation coefficients provided below.

Table 4 shows the descriptive data for the total sample (N = 768) and the correlation matrix for the three JCQ dimensions, the dimensions of the JCS, the UWES and the MBI. All Cronbach alpha scores were higher than .74.

**Table 4:** Descriptive data and correlations between the JCQ and the JCS, the UWES and the MBI.

	Range	Mean	Standard Deviation	Alpha	Task Crafting (JCQ)	Cognitive Crafting (JCQ)	Relational Crafting (JCQ)	JCQ Total
Task Crafting (JCQ)	1–6	4.76	0.76	.758				
Cognitive Crafting (JCQ)	1–6	4.51	1.08	.868	.42*			
Relational Crafting (JCQ)	1–6	4.43	0.98	.790	.35*	.37*		
JCQ Total	1–6	4.57	0.73	.880	.70*	.80*	.76*	
Increasing structural job resources (JCS)	1–7	6.14	0.67	.799	.44*	.27*	.17*	.36*
Decreasing hindering job demands (JCS)	1–7	3.53	1.09	.776	.01	.06	-.005	.03
Increasing social job resources (JCS)	1–7	4.09	1.25	.759	.20*	.22*	.29*	.31*
Increasing challenging job demands (JCS)	1–7	5.30	0.98	.748	.58*	.36*	.34*	.53*
JCS Total	1–7	4.70	0.61	.765	.42*	.33*	.31*	.45*
Vigor (UWES)	0–6	4.11	1.16	.829	.39*	.43*	.30*	.48*
Dedication (UWES)	0–6	4.40	1.27	.885	.41*	.47*	.30*	.51*
Absorption (UWES)	0–6	4.09	1.19	.749	.36*	.39*	.25*	.42*
UWES-9 total	0–6	4.20	1.08	.916	.43*	.48*	.31*	.52*
Cynicism (MBI)	0–6	1.85	1.35	.858	-.25*	-.29*	-.21*	-.33*
Emotional exhaustion (MBI)	0–6	2.31	1.38	.904	-.17*	-.18*	-.14*	-.21*
Professional efficacy (MBI)	0–6	4.75	0.79	.813	.39*	.41*	.28*	.45*

The adapted Spanish version of the JCS was used to measure concurrent validity, since its purpose is to assess the same concept as the scale presented in this paper. Correlations were statistically significant in all cases (coefficients ranging from .17 to .58), except for “decreasing hindering job demands”, which did not show significant correlations with any of the JCQ’s three dimensions.

Engagement and burnout measures were utilized to assess convergent validity, since they are commonly used to evaluate constructs that are theoretically related to job crafting. As expected, correlation coefficients were statistically significant in all cases. A positive association was found between the JCQ dimensions, the UWES total score, the UWES dimensions, and the professional efficacy in the MBI (with coefficients that ranged from .25 to .48), and negative associations were found with cynicism and emotional exhaustion in the MBI (with coefficients that ranged from -.14 to -.29).

## Discussion

This study sought to analyze the psychometric properties of the Job Crating Questionnaire [16] and to test the validity of its Spanish version on a sample of employees.

The results obtained in this study were very similar to those obtained by the authors of the original JCQ study; they exhibited adequate psychometric characteristics and good-fit-indexes. The three-factor model including task crafting (5 items), relational crafting (5 items) and cognitive crafting (5 items) remained statistically adequate, which suggests that this is an instrument with a solid structure, perfectly suitable for assessing job crafting levels among employees.

After analyzing the fit of the different models and, following Burnham and Anderson [40], a check was made to see that the AIC was less than 10. The model ultimately proposed maintains the 15 items included in the original scale, despite the fact that item 5 (“Give preference to work tasks that suit your skills or interests”) had an item-total correlation of .365, lower than the other items, and a factor loading of below .30. However, deleting this item did not enhance the reliability of the instrument (Total Cronbach’s alpha = .88), which was very similar to that found in the study conducted to validate the original scale (α = .91), and the Wald Test did not suggest that it be removed. Therefore, it was thought best to maintain the original structure and attempt to confirm it by performing studies on different samples in the future. The concurrent and convergent validity of the JCQ with other constructs was also analyzed. Regarding evidence of concurrent validity, three JCS dimensions (increasing structural job resources, increasing social job resources, and increasing challenging job demands) were found to significantly correlate with the three JCQ dimensions, though correlation coefficients were either weak or moderate (between .17 and .58). The “task crafting” dimension in the JCQ showed the strongest relationship with the JCS dimensions, as it was the closest to Tims and Bakker’ model [3] from a conceptual point of view. It should be noted that none of the JCQ dimensions exhibited a significant relationship with the “decreasing hindering job demands” dimension from the JCS. Similar results have been observed in previous studies, such as the one that validated the original JCS, in which no relationship was found between this dimension and the other three dimensions in the same scale, nor with the “personal initiative” variable [6]. Nor did the study that validated the instrument in Spanish show any significant correlations between this dimension and the proactivity measure [24]. All of the above considerations reaffirmed that there may be different job crafting models that include different dimensions. They also highlighted the need to have an adapted Spanish version of the JCS, which will enable researchers to accommodate and assess the cognitive dimension of this construct that is not taken into account in the model developed by Tims and Bakker [3]. This dimension is certainly interesting from the perspective of organizational psychology, as it makes it possible for employees to attribute meaning to their work.

As regards evidence of convergent validity, the three dimensions that make up the JCQ (task crafting, cognitive crafting and relational crafting) significantly correlated with engagement and burnout. Positive correlations were found with the three dimensions of the UWES (vigor, dedication and absorption) and the professional efficacy dimension in the MBI, whereas negative correlations were found with the cynicism and emotional exhaustion dimensions in the MBI. This distinction is consistent with the results from previous studies of burnout dimensions. Schaufeli, Salanova, González-Romá and Bakker [41] conducted a study on engagement and burnout measures and found a factor that explained burnout that only included exhaustion and cynicism, and also another factor that accounted for engagement that included its three dimensions plus the personal efficacy dimension from burnout. Later, Schaufeli and Bakker [42] demonstrated that the model that included personal efficacy within the dimensions that explained engagement showed better fit indexes among four different samples, compared to other alternative models in which the three burnout dimensions and the three engagement dimensions were presented separately. Therefore, they proposed that ideally the personal efficacy dimension should be included in the theoretical concept of engagement [42].

Moreover, various studies have correlated job crafting with variables deemed positive for work, such as engagement [43, 7, 10]. This has also been found in some longitudinal studies [11] and provides some insight into the stability of this correlation.

While the results obtained in this study were adequate, some caution needs to be taken due to its inherent limitations. Firstly, given the cross-sectional nature of the study, the psychometric characteristics of the scale cannot be analyzed in their entirety. Therefore, longitudinal studies should be conducted to confirm the stability of this scale. Secondly, it would be of interest to continue testing the proposed model and expand the study of its psychometric properties to samples with different work features and have adaptations adjusted to these contexts. And thirdly, taking into account the homogeneity of the sample, and that it included employees recruited online who had specific work characteristics in terms of employment category and level of education, validity studies should be conducted in the future using samples that include different types of employees and other populations to supplement and extend these results.

The results show that the Spanish translation of the JCQ is a suitable instrument for measuring Job Crafting, which also reinforces the factor structure proposed by the original authors and represents the conceptualization provided by Wrzesniewski and Dutton [1].

Knowledge of the concept of job crafting can be expanded further by obtaining evidence of this tool’s validity, taking into account the different theoretical proposals and gaining additional knowledge of the concept and its different dimensions. This is a relatively new concept within organizational psychology that places employees at the core of the process of change. It stresses the resources they possess to make alterations and give meaning to the tasks performed in their jobs, thus enabling them to experience higher levels of engagement and wellbeing. This directly translates into positive outcomes both for the organization and for the individual’s work environment.

The above findings reaffirm that it is important for organizations and places of work to be aware of and promote their employees’ potential to modify or adjust their beliefs about their jobs. In this way, they will be able to apply the cognitive aspects of job crafting to theirjobs, which make their employees’ work meaningful both for them and for their environment.

## Supporting information

S1 Appendix Job Crafting Questionnaire (JCQ) items. (PDF)
