# Supplementary material for: Cognitive, relational and task crafting: Spanish adaptation and analysis of psychometric properties of the Job Crafting Questionnaire
Source: PLoS One. 2019 Oct 7;14(10):e0223539. doi: 10.1371/journal.pone.0223539 (PMC6779232; doi:10.1371/journal.pone.0223539)
Supplement: S1 Appendix — (PDF) [file pone.0223539.s001.pdf]

Appendix

Job Crafting Questionnaire (JCQ) items (Slemp and Vella-Brodrick, 2013)

	Spanish
1.	Incorporar nuevos enfoques para mejorar tu trabajo
2.	Cambiar el alcance o tipo de tareas que llevas a cabo en tu trabajo
3.	Incorporar en tu trabajo nuevas tareas que crees que se ajustan mejor a tus habilidades o intereses
4.	Tomar la decisión de asumir tareas adicionales en el trabajo
5.	Dar prioridad a tareas que se ajustan a tus habilidades o intereses
6.	Reflexionar sobre cómo el trabajo da sentido a tu vida
7.	Recordarte a ti mismo/a el valor que tiene tu trabajo para el éxito de la organización
8.	Recordarte a ti mismo/a la importancia de tu trabajo para la comunidad
9.	Identificar las maneras en que tu trabajo influye positivamente en tu vida
10.	Valorar el papel que tiene tu trabajo en tu bienestar general
11.	Esforzarte por conocer bien a las personas en el trabajo
12.	Organizar actividades sociales en el trabajo (por ejemplo, celebrar el cumpleaños de un compañero/a)
13.	Organizar o asistir a eventos sociales directamente relacionados con el trabajo
14.	Tomar la decisión de actuar como mentor de las nuevas incorporaciones (ya sea de manera oficial o no)
15.	Entablar amistad con las personas del trabajo que tengan habilidades o intereses similares a los tuyos
